# MALDI-TOF-MS serum protein profiling for developing diagnostic models and identifying serum markers for discogenic low back pain

**DOI:** 10.1186/1471-2474-15-193

**Published:** 2014-06-02

**Authors:** Yin-gang Zhang, Ren-qi Jiang, Tuan-Mao Guo, Shi-Xun Wu, Wei-Juan Ma

**Affiliations:** 1 Department of Orthopedics, First Affiliated Hospital of Medical College of Xi’an Jiaotong University, Xi’an 710061, China; 2 Department of Sports Injury, Hong-Hui Hospital, Xi’an Jiaotong University College of Medicine, Xi’an 710054, China; 3 Key Laboratory of Environment and Genes Related to Diseases, Ministry of Education, Xi’an Jiaotong University, Xi’an 710061, China

**Keywords:** Chronic low back pain, Dicogenic low back pain, Lumbar disc herniation, MALDI-TOF-MS, Serum profiling, Biomarker

## Abstract

**Background:**

The identification of the cause of chronic low back pain (CLBP) represents a great challenge to orthopedists due to the controversy over the diagnosis of discogenic low back pain (DLBP) and the existence of a number of cases of CLBP of unknown origin. This study aimed to develop diagnostic models to distinguish DLBP from other forms of CLBP and to identify serum biomarkers for DLBP.

**Methods:**

Serum samples were collected from patients with DLBP, chronic lumbar disc herniation (LDH), or CLBP of unknown origin, and healthy controls (N), and randomly divided into a training set (*n* = 30) and a blind test set (*n* = 30). Matrix-assisted laser desorption ionization time-of-flight mass spectrometry was performed for protein profiling of these samples. After the discriminative ability of two most significantly differential peaks from each two groups was assessed using scatter plots, classification models were developed using differential peptide peaks to evaluate their diagnostic accuracy. The identity of peptides corresponding to three representative differential peaks was analyzed.

**Results:**

The fewest statistically significant differential peaks were identified between DLBP and CLBP (3), followed by CLBP vs. N (5), DLBP vs. N (9), LDH vs. CLBP (20), DLBP vs. LDH (23), and LDH vs. N (43). The discriminative ability of two most significantly differential peaks was poor in classifying DLBP vs. CLBP but good in classifying DLBP vs. LDH. The accuracy of models for classification of DLBP *vs.* CLBP was not very high in the blind test (forecasting ability, 67.24%; sensitivity, 70%), although a higher accuracy was observed for classification of DLBP *vs*. LDH and LDH *vs*. N (forecasting abilities, ~90%; sensitivities, >90%). A further investigation of three representative differential peaks led to the identification of two peaks as peptides of complement C3, and one peak as a human fibrinogen peptide.

**Conclusions:**

Our findings benefit not only the diagnosis of CLBP but also the understanding of the differences between different forms of DLBP. The ability to distinguish between different causes of CLBP and the identification of serum biomarkers may be of great value to diagnose different causes of DLBP and predict treatment efficacy.

## Background

Low back pain (LBP) is an extremely common musculoskeletal disorder that affects an estimated 80% of people at some time in their lives and represents the most common cause of disability [1]. Chronic LBP (CLBP) is the most frequent cause of activity limitation in adults under the age of 45 [2]. Common causes of LBP include disc degeneration, lumbar disc herniation (LDH), lumbar spinal stenosis, lumbar instability syndrome, and waist soft tissue lesions [3-5]. Clinically, only a small proportion (~20%) of LBP cases can be attributed with reasonable certainty to a pathologic or anatomical entity, because most of the signs and symptoms are not specific and are difficult to distinguish among diseases that exhibit LBP [2]. In addition, there exist a significant number of cases of CLBP of unknown origin. Therefore, an accurate diagnosis of the cause of LBP represents a great challenge to orthopedists.

Disc degeneration can cause persistent pain and is believed to be a major cause of CLBP [6]. Clinical disease resulting from this pathological process is known as discogenic low back pain (DLBP). DLBP and LDH are the two most common causes of CLBP, accounting for ~40% and ~30% of all cases of CLBP, respectively [2,7-12]. In contrast to LDH, DLBP is not accompanied by radicular symptoms or radiological evidence of excessive activity of nerves or segments [13]. Given that DLBP is clinically featured with many subjective symptoms, numerous risk factors, but has few objective positive markers and unclear pathogenesis, its diagnosis is often elusive [2]. Discography is currently the main diagnostic modality for DLBP; however, its reliability is controversial because discographic results rely heavily on the reproduction of the patient’s pain and the operator’s judgment and is therefore largely influenced by subjective factors. In addition, discography can cause many complications due to its invasive nature [2]. Thus, there is an intense debate over discography as a diagnostic tool to distinguish DLBP from other forms of CLBP and a more accurate, reliable and non-invasive diagnostic method is required.

DLBP is caused by a variety of pathologic processes, including degeneration, endplate damage and inflammation, which stimulate the intradiscal nociceptors while the disc periphery remains intact [2]. Normally there is a high density of blood vessels and nerves in the outer 1/3 of the annulus fibrosus, but not inside the disc. Degenerated intervertebral disc induces crack formation in the endplate and the outer 1/3 of the annulus fibrosus due to biomechanical reasons. As disc degeneration progresses, nascent vascular nerve endings grow inward along the fissure to form inflammatory granulation tissue that leads to the distribution of vessels and nerves inside the disc, even within the nucleus pulposus [2]. During this process, biochemical, metabolic, inflammatory and immuno-reactive products from damaged cells can enter the bloodstream and alter the blood protein spectrum [9]. Since certain serum proteins can serve as diagnostic markers for the disease that causes their release from cells [14], serum proteomic profiling of DLBP will provide a means to identify diagnostic biomarkers for this clinical entity. Identification of objective biomarkers in serum that can aid in differential diagnosis would be of great clinical benefit.

Most serum proteins, including disease biomarkers, are often present in small amounts and are difficult to detect using conventional methods [15]. Capture of low-abundance proteins is critical for facilitating marker discovery. Bead-based fractionation can greatly enrich low-abundance proteins and selectively separate certain peptides according to different chemical chromatographic surfaces on the outer layer of magnetic beads. The combination of bead-based fractionation with matrix-assisted laser desorption ionization time-of-flight mass spectrometry (MALDI-TOF-MS) has been widely used for disease detection and biomarker identification using human body fluids (e.g., blood and urine), and carries advantages such as minimal invasiveness, high efficiency, low cost, and easy manipulation [16]. The more sophisticated techniques, such as Fourier transform ion cyclotron resonance tandem mass spectrometry (MS/MS) and linear trap quadrupole orbitrap MS, allow the direct identification of peptide sequences. Currently, there have been no reports of the use of these techniques for disease detection and biomarker identification in patients with DLBP.

In the current study, we conducted a MALDI-TOF-MS analysis of serum samples from patients with DLBP, chronic LDH, or CLBP of unknown origin, and normal controls, with an aim to establish diagnostic models for classification of these different entities and identify serum biomarkers for differentiation of DLBP from other forms of LBP.

## Methods

### Patients and serum sample collection

This study included three groups of patients: those with DLBP (*n* = 60), CLBP of unknown origin (*n* = 60), or chronic LDH (*n* = 60). All of them were in-patients from the Department of Orthopedics of the Affiliated Hospital of Xi’an Jiaotong University or from the Department of Spine Surgery of the Xi'an Garden City Hospital. Sixty healthy volunteers were recruited as normal controls (N). Different groups were matched for race, age and geographic region. All subjects underwent MRI and CT examinations and pain evaluation using the visual analogue scale (VAS) and Oswestry disability index (ODI) (Table 1). Imaging data for each subject were confirmed by at least two physicians and a radiologist. Patients who were suspected of having DLBP based on clinical symptoms and imaging results received further discographic examinations. All study subjects gave their informed consent. The study protocol was approved by the Ethics Committee of the Xi'an Jiaotong University School of Medicine.

**Table 1:** Clinical data for subjects of different groups

	**Group**	**Case (n)**	**Age (years)**	**VAS value**	**ODI value**
Training set	DLBP	30	42.81 ± 13.37	6.70 ± 1.86	20.60 ± 8.60
	CLBP	30	43.05 ± 15.21	5.48 ± 3.61	25.37 ± 11.24
	LDH	30	42.58 ± 9.67	7.21 ± 2.37	31.45 ± 9.07
	N	30	41.94 ± 7.65	—	—
Blind test set	DLBP	30	43.21 ± 12.89	6.90 ± 1.49	21.30 ± 8.78
	CLBP	30	41.35 ± 15.43	5.32 ± 3.51	26.21 ± 10.89
	LDH	30	43.32 ± 10.21	7.61 ± 2.42	32.21 ± 9.35
	N	30	42.84 ± 9.98	—	—

Data shown are mean ± standard deviation. DLBP: discogenic low back pain; CLBP: chronic low back pain of unknown origin; LDH: lumbar disc herniation; N: normal controls; VAS: visual analogue scale; ODI: Oswestry disability index.

Fasting venous blood samples (5 mL) were drawn from each subject and placed in vacuum blood collection tubes containing 2% EDTA-K3. The samples were then centrifuged at 272 g for 10 minutes at 10°C, and the sera were collected and stored at -80°C for further use.

### Inclusion and exclusion criteria

Inclusion criteria for DLBP were (i) recurrent pain in the lower back, buttocks, and greater trochanter for >6 months, which was aggravated after activity, prolonged sitting or standing; (ii) a negative straight leg raising test and no lumbar tenderness or nerve root damage; (iii) normal X-ray, CT results, and normal MRI results or the presence of “dark disc”, “high imaging zone” or “modic change” [2]; and (iv) discographic results revealing abnormal morphology of the disc and induction of consistent pain, without a pain response in the adjacent control disc. Exclusion criteria for DLBP were (i) serious primary diseases of the cardiovascular, digestive, urinary, hematopoietic or endocrine systems; (ii) mental disorders and hysteria; (iii) disc injury caused by a one-time severe trauma; and (iv) a positive iodine allergy test.

Inclusion and exclusion criteria for CLBP of unknown origin were (i) LBP for >6 months; (ii) negative discographic results or radiological results ruling out chronic LDH and degenerative pain caused by the intervertebral disc; and (iii) no spinal or sacroiliac joint inflammation, cancer, tuberculosis, deformities, or other organic lesion. Patients with visceral LBP were also excluded.

Inclusion criteria for chronic LDH were (i) LBP for >6 months; (ii) recurrent LBP radiating down the leg, with or without signs of cauda equina compression; (iii) postural scoliosis, with tenderness in the interspinal lesion and possible radiating pain caused by pressure on the sides of spinous processes; (iv) limited spine flexion, a positive straight leg raising test and augmentation test; (v) sensory dysfunction, decreased muscle strength, and abnormal reflexes; (vi) X-ray results showing reduction or disappearance of physiological curvature of the lumbar spine and narrowed intervertebral space, and CT or MRI results revealing the compression of nerves or segments by the disc and excluding spinal tuberculosis, cancer and other diseases. LDH was diagnosed when (i), (vi) and at least two of (ii) - (v) were met.

### Experimental design

Serum specimens collected from each group were randomly divided into a training set (*n* = 30) and a blind test set (*n* = 30). The training set was analyzed first, followed by a blind test set to assess model performance and to qualify biomarkers. The test set was blinded to personnel processing the samples and analyzing the data.

### WCX magnetic bead analysis

Serum samples were placed at 4°C for 2 hours, centrifuged at 1800 g rpm at 4°C for 10 minutes, aliquoted and stored at -80°C. Peptides were isolated from the serum using a magnetic beads-based weak cation exchange (MB-WCX) kit (Bruker Daltonics, Bremen, Germany) according to the manufacturer’s instructions. Magnetic beads with C8-functionality were divided into 5-μL aliquots in a 96-well microtiter plate, which was placed on the magnetic beads separation device (MPC-auto96, Dynal, Oslo, Norway) with the magnet down. Ten microliters of MB-WCX binding solution and 5 μL of serum sample were added to the beads and carefully mixed using the mixing feature of the robot. The sample was incubated for 30 seconds and the magnet was lifted, followed by a 30-second waiting interval to settle the magnetic beads. The supernatant was removed and the magnet was lowered again. The magnetic beads were washed three times with MB-WCX washing solution provided with the kit, lifting and lowering the magnet as required. The peptides were eluted from the beads with 10 μL of 50% acetonitrile (ACN) and 2 μL of the elute was transferred to a fresh 348-well microtiter plate (Greiner, Frickenhausen, Germany). The remaining elute (6 μL) was transferred to an auto sample vial containing 54 μL of water and stored for later use. Fifteen microliters of a-cyano-4-hydroxycinnamic acid (0.3 g/L in ethanol:acetone 2:1) was added to the 1-μL elute in the 348-well microtiter plate and mixed carefully. One microliter of this mixture was spotted in quadruplicate on a MALDI AnchorChipTM (Bruker Daltonics).

### MALDI-TOF MS

MALDI-TOF-MS measurements were performed using an Ultraflex TOF/TOF instrument (Bruker Daltonics) equipped with a SCOUT ion source, operating in linear mode. Ions formed with a N2 pulse laser beam (337 nm) were accelerated to 25 kV. For MALDI-TOF-MS analysis, 1 μL of the above diluted purified serum peptide was mixed with 0.5 μL of matrix solution and allowed to dry onto the MALDI sample plate (600 μM AnchorChip™, Bruker Daltonics) according to the manufacturer’s instructions. Two peptides were also included in the matrix solution for internal calibration: 10 pmol/mL angiotensin II and 10 pmol/mL ACTH18-39 (Bruker Daltonics). Laser desorption was targeted randomly on the sample plate and samples were measured using an Autoflex III MALDI-TOF mass spectrometer (Bruker Daltonics), operating in positive ion linear (reflection) mode. Ionization was achieved by irradiation with a 50 Hz nitrogen laser. Spectra were the mean of 100 ionizations with fixed laser power in linear geometry mode and mass maps were obtained in reflectron mode. The spectra were calibrated externally with a mixture of protein/peptide standards in the range of 1,000 to 12,000 Da (Bruker Daltonics). The criteria for peak detection were: signal/noise ratio >5.2 Da peak width filter, and maximum peak number of 200. For data bank analysis, all spectra were processed by automatic baseline subtraction, peak detection, recalibration, and peak area calculation according to the predefined parameter settings. The intensities of the peaks of interest were normalized against the peak intensity of the ACTH internal standard. These mass shifts were corrected by the FlexAnalysis™ software after alignment with the two internal standards.

### Identification of differential peptides

Peptide extracts were dried and re-suspended in 15–20 mL of 5% formic acid for further MS/MS analysis using a LTQ mass spectrometer (Thermo Fisher Scientific, Waltham, USA). Typically, 5 μL of peptide extracts were actually injected for analysis. For LTQ mass spectrometer analysis, the peptide extracts were loaded at 15 mL/min for 6 minutes on a nanoAcquity™ column, followed by eluting and separating on a nanoAcquity™ UPLC™ column, using 90 minute gradients with 95% water, 5% ACN, 0.1% formic acid (solvent A); and 95% ACN, 5% water, 0.1% formic acid (solvent B) at a flow rate of 300 nL/min. The samples were run in data-dependent mode, where each full MS scanning was followed by three consecutive MS/MS scans of the three most abundant peptide molecular ions (typically doubly and triply charged ions), which were selected consecutively for collision-induced dissociation. The MS survey scans (300–2,000 Da) were carried out and the acquisition cycle consisted of a survey scan at the highest resolving power (100,000). Dynamic exclusion was used with a series of parameters and the acquired MS/MS data were processed using BioworksBrowser 3.3.1. A sequence database search was performed with the International Protein Index (IPI Human3.45).

### Statistical analysis

All MALDI-TOF-MS spectra were analyzed with FlexAnalysis™ to detect the peak intensities of interest and CLINPROT™ software to compile the peaks across the spectra obtained from all samples (Bruker Daltonics). All statistical comparisons were performed using SPSS software version 13.0 (SPSS Inc., Chicago, IL, USA). The non-parametric Wilcoxon test or Kruskal-Wallis test (>2 classes) was used to compare two peptide peaks, and the Kruskal-Wallis test was used to compare three or more peptide peaks. *P*-values < 0.05 were considered statistically significant. Neural network algorithm (SNN), fast classification algorithm (QC) and genetic algorithm (GA) methods were used to establish specific differences in the protein diagnostic model and to predict the diagnostic accuracy. The models based on peptide peaks showing different peak area were developed, and the best of the three models was presented.

## Results

### Detection of differential peptide peaks

In the pair-wise comparisons, processing of MALDI-TOF spectra resulted in the identification of 3 differential peptide peaks between DLBP and CLBP of unknown origin, 23 between DLBP and LDH, 9 between DLBP and N, 20 between LDH and CLBP of unknown origin, and 43 between CLBP of unknown origin and N and 5 LDH vs. N. Of note, although there were 95 peaks showing different peak area between DLBP and CLBP, only three (1741 m/z, 1898 m/z, and 5754 m/z) of them showed statistically significant differences. The first two peaks were up-regulated and the third one was down-regulated in DLBP. All these peptide peaks, their peak mass, *P* values, average peak intensity between two groups and standard deviation (SD) are shown in Additional file 1 (The character of different peaks between experimental groups).

### Discriminative ability of representative differential peptide peaks

**Figure 1:**
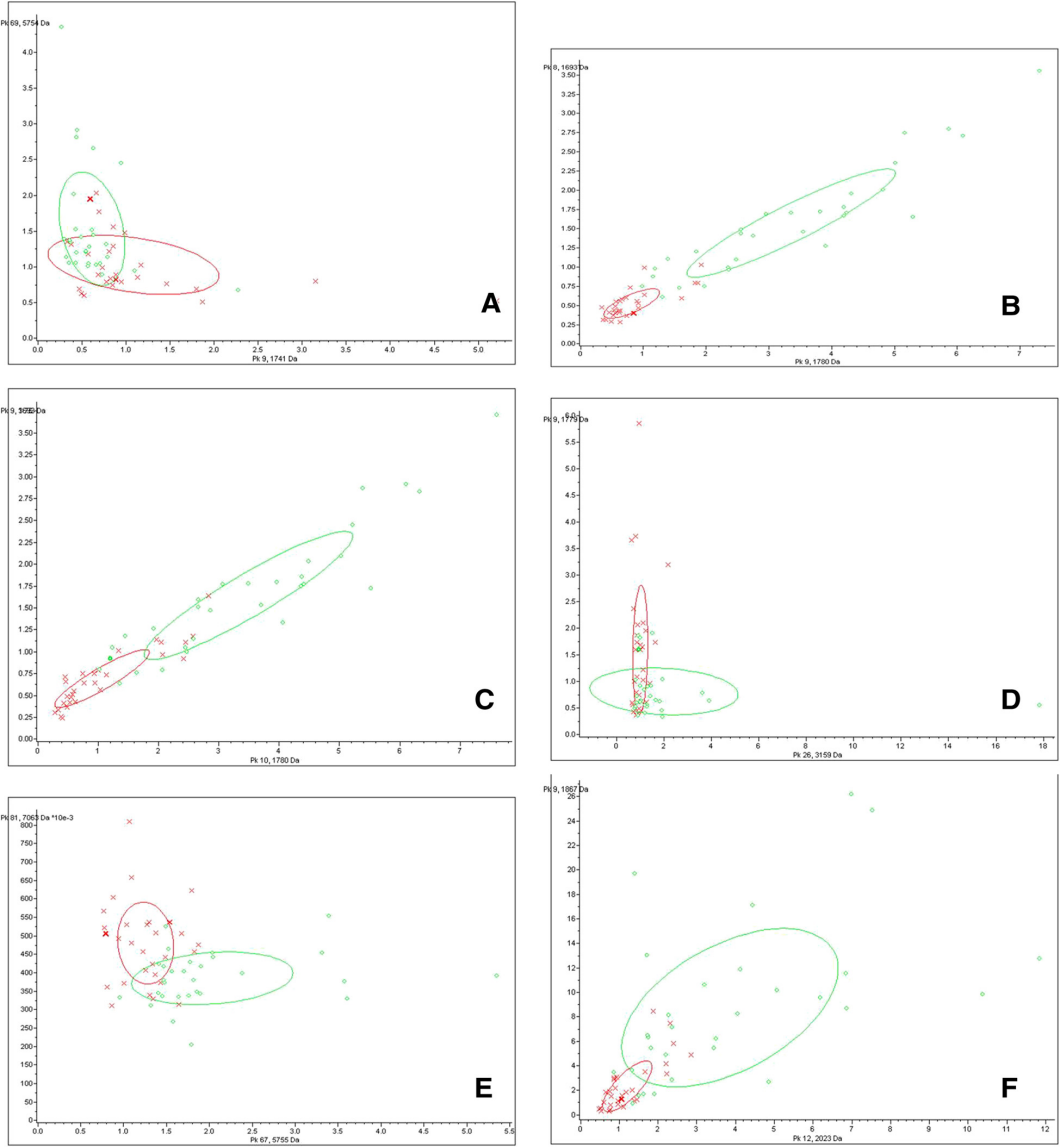
**Scatter plots showing the discriminative ability of representative differential peptide peaks.** DLBP vs. CLBP of unknown origin **(A)**, DLBP vs. LDH **(B)**, DLBP vs. N **(C)**, CLBP of unknown origin vs. LDH **(D)**, CLBP of unknown origin vs. N **(E)**, and N vs. LDH **(F)** were discriminated using peaks at 1741 m/z and 5754 m/z, those at 1779 m/z and 1692 m/z, 3159 m/z and 1779 m/z, 1779 m/z and 1692 m/z, 5755 m/z and 7062 m/z, and 2023 m/z and 866 m/z, respectively. The x-axis and y-axis represent the abundance of each peptide (× for DLBP, ○ for CLBP).

To examine the ability of differential peptide peaks to classify different groups of subjects, we selected the two most significantly differential peaks from each two groups and displayed them in scatter plots (Figure 1). A large overlapping area between DLBP and CLBP sample distributions indicated that the two peaks (1741 m/z and 5754 m/z) were poor at classifying DLBP and CLBP (Figure 1A). In contrast, there was little overlap between DLBP and LDH sample distributions, indicating that the two peaks (1779 m/z and 1692 m/z) were good at classifying these two groups (Figure 1B). The ability of other peaks to classify other groups fell between these two sets of peaks (Figure 1C-F).

### Development of diagnostic models and evaluation of their diagnostic accuracy

To determine whether multiple peaks could accurately classify different groups of subjects, models based on peptide peaks showing different peak area were developed using GA, SNN and QC. These classification models were tested for their recognition capability and forecasting ability, followed by a blind test to calculate their sensitivity and specificity as diagnostic classification models. Paired comparison analyses showed that the classification model for DLBP vs. CLBP had the lowest accuracy, with a recognition capability of 82.66%, a forecasting ability of 67.24%, and a sensitivity of 70% in the blind test (Table 2). The classification models for DLBP vs. LDH and LDH vs. N were more accurate, with forecasting abilities of nearly 90% and sensitivities greater than 90% in the blind test.

**Table 2:** Diagnostic accuracy of different classification models

**Comparison**	**Peaks included in the classification model (m/z)**	**Training set**		**Blind test set**	
		**Recognition capability**	**Forecasting ability**	**Sensitivity**	**Specificity**
DLBP vs. CLBP	6935.16, 1262.74, 9290.15, 5533.89, 4396.51, 5132.44, 2863.16, 1866.76, 6270.64, 4054.52, 8141.73, 4468.72, 7192.08, 1982.17, 2953.54	82.66%	67.24%	70%	63.33%
DLBP vs. LDH	1692.71,1779.79	88.33%	86.67%	93.33%	80%
DLBP vs. N	1779.28, 3159.06, 5292.71, 5754.98, 4054.22, 5247.91, 6934.55, 4527.3, 7473.04, 3883.04, 6270.34, 3449.05, 5265.25, 4395.98, 5131.5	100%	74.71%	86.67%	83.3%
LDH vs. CLBP	1692.72, 1779.74, 7766, 4121.69, 2933.47	94.89%	81.44%	93.33%	86.67%
LDH vs. N	1866.86, 2023.1, 3263.09, 4527.38, 6938.15, 7639.28, 5635.59, 6028.38	95%	86.67%	96.67	93.33%
CLBP vs. N	5755.18, 4964.69, 1450.69, 5629.16, 4018.94, 2790.29, 4467.25, 9184.37, 7062.96, 5293.26	95%	76.32%	73.33%	73.33%
DLBP + LDH vs. N	7923.02, 2933.11, 2790.11, 6562.89, 5292.65, 1450.6, 4054.08, 5755.42	95.82%	73.28%	95%	96.67%
DLBP + LDH vs. CLBP	1866.85, 1897.7, 8141.79, 5132.49, 5807.7, 5533.54, 4362.7, 3192.61	90.56%	70.02%	90%	90%
DLBP + CLBP + LDH vs. N	5755.08, 7061.97, 2790.07, 5247.76, 1450.59, 8142.27	88.86%	73.83%	86.67%	86.67%

DLBP: discogenic low back pain; CLBP: chronic low back pain of unknown origin; LDH: lumbar disc herniation; N: normal controls.

We next combined the DLBP and LDH groups as a disc degeneration group (DLBP + LDH) and compared it with group N using the above-mentioned algorithms. This resulted in a recognition capability of 95.82%, a forecasting ability of 73.28%, and a sensitivity as high as 95% in the blind test. When combining the DLBP, CLBP, and LDH groups as a LBP group (DLBP + CLBP + LDH) and comparing it with group N, we obtained a recognition capability of 88.86%, a forecasting ability of 73.83%, and a sensitivity of 86.67% in the blind test. Both combination groups could be classified from group N accurately (Table 2).

### Identification of differential peptides

**Figure 2:**
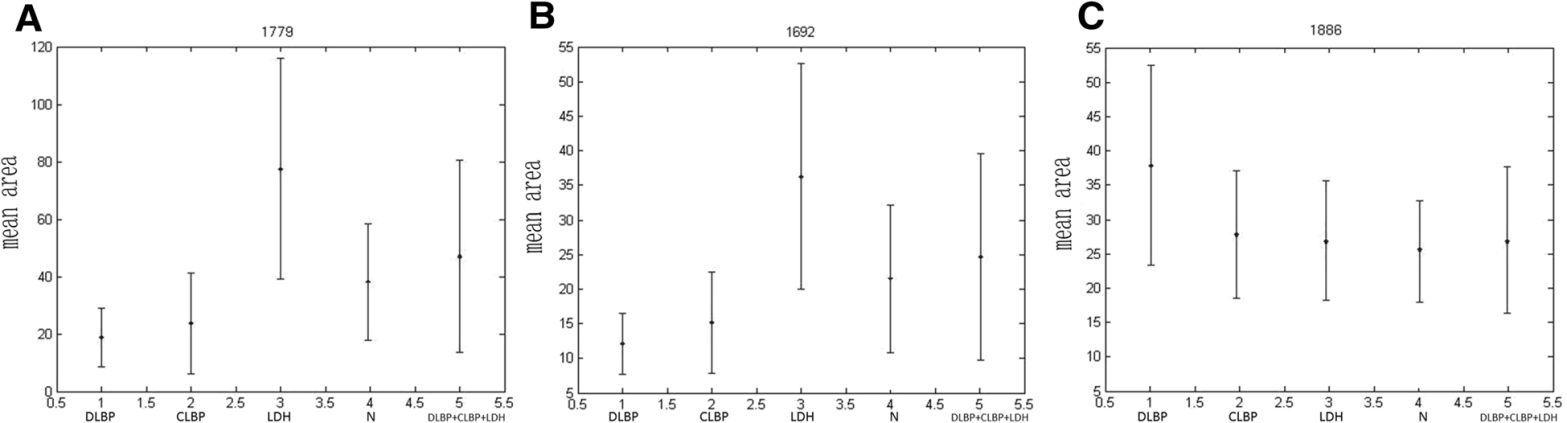
Comparison of the mean areas of the peaks at 1779 m/z (A), 1692 m/z (B), and 1886 m/z (C) among different groups.

**Figure 3:**
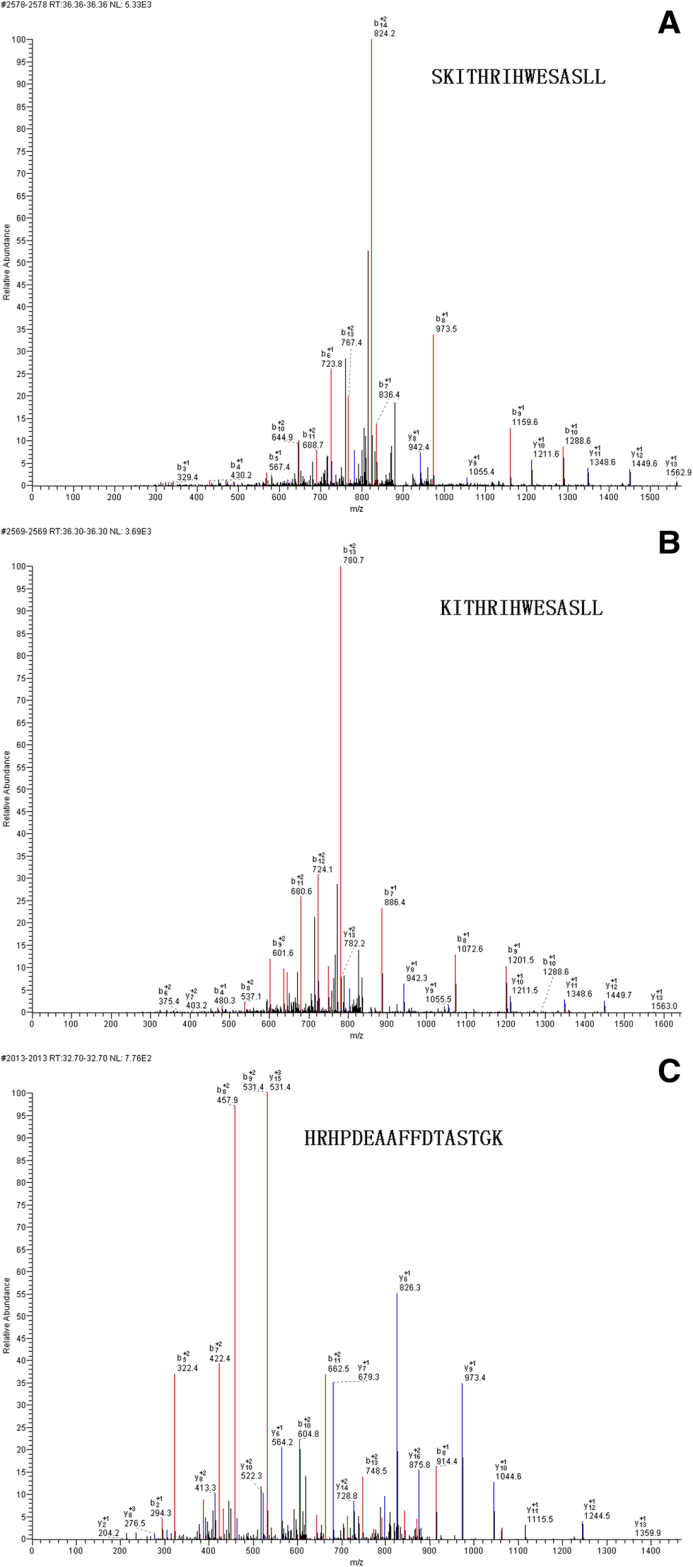
**MS/MS identification of selected serum peptides as fragments of complement C3 (A) and (B), and isoform 1 of fibrinogen alpha chain precursor (C).** The fragment ion spectra shown were taken for a MS/MS ion search of Protein Knowledgebase (UniProKB) (http://www.uniprot.org). b and y fragment ion series are indicated together with the limited sequences.

Protein/peptide markers were chosen based on the following considerations: (i) significantly differentially expressed peptides, especially those exhibiting a time- or dose-dependent pattern; and (ii) peptides that were able to accurately classify the control group and disease groups. Taking these into account, three differential peptides (1692.71 m/z, 1886.86 m/z and 1779.28 m/z) were selected for peptide identification. By comparing areas of the selected peptide peaks, we found that the peptides to which 1779 m/z and 1692.71 m/z corresponded showed similar expression patterns (Figure 2), with the lowest expression level in DLBP, followed by CLBP, N and LDH. Their expression levels differed significantly different between DLBP and the other three groups (*Ps* < 0.05 for all), but not between CLBP of unknown origin, LDH and N. The peptide peak at 1886 m/z peptide had the largest area in DLBP, followed by CLBP of unknown origin, LDH and N. Its expression level also differed significantly between DLBP and the other three groups (*P* < 0.05), but not between CLBP of unknown origin, LDH and N. MS/MS analysis of the peaks at 1779 and 1692 m/z detected most b and y ions (Figure 3A and B) and the sequences of these two peptides were S.SKITHRIHWESASLL.R and S.KITHRIHWESASLL.R, which corresponded to complement C3 and complement C3 precursor, respectively. The peak at 1886 m/z was analyzed by MS/MS and the sequence of this peptide was identified as R.HRHPDEAAFFDTASTGK.T, which is unique to fibrinogen alpha chain precursor (Figure 3C).

## Discussion

The diagnosis of the cause of LBP represents a great challenge to orthopedists due to the controversy over the diagnosis of DLBP and the existence of a significant number of cases of CLBP of unknown origin [2]. There is currently an urgent need to discover biomarkers for diagnosis of DLBP and classify it from other forms of CLBP. In the present study, we used the MALDI-TOF-MS technology to perform protein profiling of serum samples from patients with DLBP, chronic LDH, or CLBP of unknown origin, and healthy controls. By paired comparison analysis of protein peaks between the four groups of subjects, we identified a series of peaks showing significant differential expression between different groups. We then compared the discriminative ability of representative differential peptide peaks to classify different groups of subjects and constructed diagnostic models using peptide peaks showing different peak area to evaluate their diagnostic accuracy. We found that the accuracy of classification of DLBP *vs.* CLBP was not very high in the blind test (forecasting ability, 67.24%; sensitivity, 70%), although a higher accuracy was observed for classification of DLBP *vs*. LDH and LDH *vs*. N (forecasting abilities, ~90%; sensitivities, >90%). Finally, we pursued a further investigation of three representative differential peaks and identified the peaks at 1779 m/z and 1692 m/z as peptides of complement C3, and the peak at 1886 m/z as a human fibrinogen peptide. These results benefit not only the diagnosis of CLBP but also the understanding of the differences of different forms of CLBP. The ability to distinguish between different causes of LBP and the identification of serum biomarkers may be of great value in diagnosing different causes of DLBP and predict treatment efficacy.

The primary purpose of this study was to distinguish DLBP from CLBP of other causes and identify serum markers for DLBP. However, the fewest statistically differential peptide peaks were detected between DLBP and CLBP of unknown origin, and the diagnostic model developed based on differential peptides had the lowest accuracy in the blind test (sensitivity, 70%), although more statistically differential peptide peaks were detected and more accurate diagnostic models developed in other pairwise comparison analyses. In patients with CLBP (including those with DLBP), pain can be caused by multiple factors, including both discogenic factors and factors outside the spinal canal [17]. Since current diagnosis of DLBP relies on discography, its low sensitivity might have resulted in the misdiagnosis of some cases of DLBP as CLBP [18]. This may be the main reason for poor performance of models for classification of DLBP vs. CLBP in this study.

In our study, two differential peaks (1779 m/z and 1692 m/z) were identified as peptides of complement C3. Complement C3 is a central molecule in the complement system and plays a very important role in inflammatory and immune responses [19]. Interestingly, the complement system has been implicated in cartilage degradation, and C3 has been found to be aberrantly increased in synovial fluids from individuals with osteoarthritis [20]. However, serum levels of complement C3 were decreased in our patients with DLBP. The clinical implications of this finding remain unclear. Given that complement C3 levels are significantly decreased in patients with different types of autoimmune diseases [21,22], and that inflammation secondary to an autoimmune response to the nucleus pulposus has been implicated as a primary pain source in DLBP [23], we speculate that dysregulation of the complement system has a key role in the pathogenesis of DLBP.

Fibrinogen is a 340 kDa glycoprotein composed of three polypeptide chains. It is a major player in the mediation of inflammatory responses and inhibition of nerve fiber growth and tissue repair processes [24]. Our finding that serum levels of fibrinogen were significantly increased in patients with DLBP indicates a close relationship between fibrinogen and DLBP. Since the development of DLBP involves inflammation, nerve growth and tissue repair, it appears reasonable to surmise that elevated expression of fibrinogen may be involved in the pathogenesis of DLBP. Future studies are required to address the precise role of fibrinogen in the pathogenesis of DLBP.

Both DLBP and LDH are degenerative disc diseases. In our study, paired comparison analysis led to the identification of 23 significantly differential peaks between DLBP and LDH. The diagnostic models developed based on these differential peaks showed a good classification performance between the two groups. These findings suggest that DLBP and LDH appear not to share a similar pathogenesis. Consistent with this, some DLBP patients suffered pain for more than 300 months but did not develop LDH until the recent attack.

## Conclusions

In conclusion, we have demonstrated that the ClinProt system is effective in screening differential serum peptides between different forms of CLBP and searching for markers to develop diagnostic models for these diseases. The identification of complement C3 and fibrinogen as two potential serum biomarkers for DLBP will shed light on the further understanding of the pathogenesis of DLBP. In addition, the presence of a significant number of differential peaks between DLBP and LBP suggests they appear not to share a similar pathogenesis. However, there is still a gap between our findings and their application in clinical practice. Future multi-center, controlled studies with larger sample sizes are required to determine the sensitivity and specificity of the two identified serum biomarkers in the diagnosis of DLBP.

## Abbreviations

ACN: Acetonitrile; CLBP: Chronic low back pain; GA: Genetic algorithm; LBP: Low back pain; LDH: Lumbar disc herniation; MALDI-TOF-MS: Matrix-assisted laser desorption ionization time-of-flight mass spectrometry; MB-WCX: Magnetic beads-based weak cation exchange; MS/MS: Tandem mass spectrometry; N: Normal controls; ODI: Oswestry disability index; QC: Fast classification algorithm; SD: Standard deviation; S/N: Signal/noise; SNN: Neural network algorithm; VAS: Visual analogue scale.

## Competing interests

All authors declared there were no conflict interests involved.

## Authors’ contributions

YGZ made substantial contributions to the conception and design, acquisition of data, analysis and interpretation of data, and drafting of the manuscript; TMG carried out the WCX magnetic bead analysis and MALDI-TOF MS. RQJ participated in the design of the study and performed the statistical analysis. SXW, and Ma WJM participated in the design and coordination of the study, and assisted with drafting the manuscript. All authors carried out the analyses, read, and approved the final manuscript.

## Author’s information

Ren-qi Jiang co-first author.

## Pre-publication history

The pre-publication history for this paper can be accessed here:

http://www.biomedcentral.com/1471-2474/15/193/prepub

## Supplementary Material

Additional file 1: Table S1 The character of different peaks between experimental groups. Click here for file
