# Supplementary material for: MALDI-TOF-MS serum protein profiling for developing diagnostic models and identifying serum markers for discogenic low back pain
Source: BMC Musculoskelet Disord. 2014 Jun 2;15:193. doi: 10.1186/1471-2474-15-193 (PMC4061098; doi:10.1186/1471-2474-15-193)
Supplement: Additional file 1: Table S1 — The character of different peaks between experimental groups. [file 1471-2474-15-193-S1.doc]

Additional file 1. The character of different peaks between experimental groups

|  | Mass | P value | Ave 1 | Ave 2 | SD 1 | SD 2 |
| --- | --- | --- | --- | --- | --- | --- |
| DLBP vs. CLBP | 1741.19 | 0.0236 | 24.00 | 13.59 | 21.41 | 8.24 |
|  | 5754.25 | 0.0332 | 22.72 | 33.83 | 9.12 | 17.49 |
|  | 1897.73 | 0.0382 | 94.13 | 34.88 | 161.48 | 23.96 |
| DLBP vs. LDH | 1779.79 | < 0.000001 | 18.89 | 77.67 | 10.29 | 38.48 |
|  | 1692.71 | < 0.000001 | 12.2 | 36.4 | 4.44 | 16.33 |
|  | 1866.93 | < 0.000001 | 36.61 | 208.03 | 29.52 | 152.67 |
|  | 2023.16 | 0.00000255 | 31.35 | 91.1 | 20.07 | 65.97 |
|  | 1450.95 | 0.0000696 | 10.86 | 21.1 | 5.25 | 10.13 |
|  | 1350.24 | 0.000264 | 9.71 | 15.3 | 3.52 | 6.61 |
|  | 7192.73 | 0.00148 | 12.84 | 9.94 | 4.02 | 2.74 |
|  | 7922.37 | 0.00434 | 37.19 | 24.88 | 18.78 | 11.37 |
|  | 7637.94 | 0.00812 | 27.21 | 17.97 | 15.48 | 7.85 |
|  | 7565.17 | 0.00982 | 17.35 | 11.72 | 9.38 | 4.38 |
|  | 7765.14 | 0.0106 | 730.34 | 441.12 | 415.93 | 256.54 |
|  | 8141.06 | 0.0149 | 63.29 | 37.28 | 38.38 | 22.29 |
|  | 8603.36 | 0.0149 | 49.35 | 25.73 | 79.82 | 23.91 |
|  | 7434.15 | 0.0199 | 13.31 | 11.35 | 2.87 | 2.99 |
|  | 9288.81 | 0.0199 | 224.02 | 147.93 | 114.59 | 94.42 |
|  | 9181.52 | 0.0214 | 10.36 | 8 | 3.45 | 2.78 |
|  | 7018.73 | 0.0214 | 15.71 | 13.47 | 3.72 | 3.52 |
|  | 7473 | 0.0214 | 6.93 | 5.88 | 1.75 | 1.31 |
|  | 1886.26 | 0.035 | 37.56 | 25.89 | 14.23 | 9.64 |
|  | 7833.25 | 0.0383 | 42.46 | 30.19 | 23.76 | 15.57 |
|  | 9062.1 | 0.0383 | 8.82 | 7.08 | 2.99 | 3.04 |
|  | 6030.27 | 0.0402 | 8.02 | 10.03 | 3.86 | 4.04 |
|  | 5375.65 | 0.0403 | 9.18 | 11.42 | 3.08 | 3.3 |
| DLBP vs. N | 3159.06 | < 0.000001 | 46.11 | 23.39 | 72.58 | 7.26 |
|  | 1779.28 | < 0.000001 | 18.9 | 36.92 | 10.21 | 28.20 |
|  | 2790.19 | 0.00000255 | 25.38 | 42.22 | 24.10 | 26.95 |
|  | 1450.49 | 0.000264 | 11.01 | 23.14 | 5.25 | 18.50 |
|  | 1692.32 | 0.00148 | 11.99 | 21.43 | 4.37 | 15.15 |
|  | 7062.16 | 0.00434 | 10.08 | 12.25 | 2.18 | 2.68 |
|  | 5080.37 | 0.00812 | 7.81 | 9.86 | 2.66 | 2.64 |
|  | 5247.91 | 0.00982 | 12.91 | 15.18 | 3.15 | 2.89 |
|  | 6934.55 | 0.0106 | 5.04 | 6.32 | 1.09 | 1.83 |
| LDH vs. CLBP | 1779.74 | < 0.000001 | 77.57 | 23.68 | 38.41 | 17.43 |
|  | 1692.72 | < 0.000001 | 36.5 | 15.42 | 16.27 | 7.36 |
|  | 1866.95 | 0.00000166 | 208.15 | 52.63 | 152.72 | 63.3 |
|  | 1887.2 | 0.000014 | 56.54 | 26.5 | 32.77 | 11.38 |
|  | 2023.17 | 0.0000422 | 91.12 | 32.95 | 66.04 | 26.06 |
|  | 1450.98 | 0.000294 | 21.1 | 11.02 | 10.15 | 5.97 |
|  | 8142.19 | 0.000852 | 37.05 | 55.79 | 22.14 | 19.74 |
|  | 5532.5 | 0.000926 | 14 | 20.55 | 2.9 | 9.28 |
|  | 1350.1 | 0.00155 | 14.38 | 8.92 | 6.47 | 3.55 |
|  | 6563.39 | 0.00171 | 11.22 | 13.59 | 2.51 | 2.15 |
|  | 7766 | 0.00247 | 439.98 | 663.4 | 255.99 | 234.32 |
|  | 7565.51 | 0.00426 | 12.43 | 16.05 | 4.61 | 4.29 |
|  | 7923.17 | 0.00445 | 26.27 | 36.59 | 11.77 | 13.01 |
|  | 7191.39 | 0.00889 | 8.08 | 9.67 | 2.4 | 1.91 |
|  | 1897.73 | 0.0144 | 53.89 | 32.27 | 35.8 | 22.68 |
|  | 7431.35 | 0.0151 | 7.91 | 9.37 | 1.99 | 1.98 |
|  | 3279.99 | 0.0319 | 91.75 | 73.54 | 26.61 | 39.33 |
|  | 5064.84 | 0.0319 | 19.24 | 25.51 | 8.62 | 12.77 |
|  | 7638.59 | 0.0331 | 17.38 | 21.16 | 7.58 | 5.91 |
|  | 5248.43 | 0.0331 | 12.24 | 14.2 | 2.08 | 3.39 |
| LDH vs. N | 2023.1 | 0.00000217 | 28.57 | 91.09 | 65.95 | 15.07 |
|  | 1866.86 | 0.00000688 | 56.14 | 207.96 | 152.61 | 48.94 |
|  | 8142.51 | 0.0000069 | 69.61 | 37.09 | 22.18 | 27.76 |
|  | 1887.06 | 0.0000114 | 26.48 | 55.11 | 31.86 | 9.91 |
|  | 7923.57 | 0.0000518 | 40.96 | 25.68 | 11.55 | 13.81 |
|  | 7565.97 | 0.000061 | 17.5 | 11.98 | 4.47 | 4.77 |
|  | 1779.6 | 0.0000696 | 36.79 | 77.42 | 38.38 | 28.25 |
|  | 7639.28 | 0.0000696 | 17.72 | 11.56 | 5.38 | 4.64 |
|  | 7192.87 | 0.000221 | 7.79 | 6.08 | 1.89 | 1.56 |
|  | 7020.49 | 0.000231 | 11.83 | 9.46 | 2.55 | 2.24 |
|  | 9063.25 | 0.000263 | 18.92 | 13.03 | 4.53 | 7.48 |
|  | 1692.59 | 0.000349 | 21.61 | 36.32 | 16.36 | 15.21 |
|  | 7432.95 | 0.000372 | 10.74 | 8.68 | 2.34 | 2.02 |
|  | 7766.17 | 0.000568 | 672.89 | 440.19 | 255.94 | 254.12 |
|  | 7834.35 | 0.000568 | 39.55 | 27.07 | 14.23 | 16.17 |
|  | 5248.09 | 0.000568 | 14.88 | 12.46 | 2.13 | 2.83 |
|  | 7061.82 | 0.000656 | 11.15 | 8.54 | 2.13 | 2.52 |
|  | 3883.01 | 0.000757 | 56.5 | 35.1 | 17.33 | 27.39 |
|  | 6563.2 | 0.00166 | 12.42 | 10.37 | 2.35 | 1.93 |
|  | 9184.99 | 0.00241 | 11.15 | 8.64 | 3.17 | 2.96 |
|  | 2790.19 | 0.00259 | 42.58 | 24.66 | 18.8 | 26.91 |
|  | 4964.21 | 0.00263 | 13.02 | 9.26 | 3.74 | 5.41 |
|  | 6938.15 | 0.00299 | 5.66 | 4.5 | 0.96 | 1.54 |
|  | 8937.18 | 0.00383 | 262.53 | 118.65 | 122.98 | 218.54 |
|  | 8606 | 0.00435 | 36.96 | 26.07 | 24.06 | 32.94 |
|  | 9291.45 | 0.00442 | 222.16 | 149.72 | 95.13 | 100.27 |
|  | 4054.26 | 0.00429 | 86.65 | 59.87 | 26.04 | 32.95 |
|  | 5524.1 | 0.0191 | 13.06 | 15.06 | 2.73 | 2.82 |
|  | 4527.38 | 0.00703 | 16.04 | 19.61 | 3.68 | 4.66 |
|  | 5081.03 | 0.00947 | 7.73 | 9.54 | 2.41 | 2.57 |
|  | 4467.11 | 0.0132 | 38.65 | 64.22 | 20.23 | 37.66 |
|  | 5755.94 | 0.0132 | 21.91 | 16.21 | 8.19 | 4.54 |
|  | 3158.91 | 0.0156 | 27.27 | 22.43 | 8.78 | 7.19 |
|  | 5337.15 | 0.0213 | 148.15 | 192.67 | 51.65 | 64.24 |
|  | 5483.35 | 0.0213 | 8.67 | 10.51 | 2.24 | 2.81 |
|  | 3279.95 | 0.0293 | 91.46 | 75.08 | 26.52 | 27.57 |
|  | 3209.43 | 0.0293 | 74.69 | 63.59 | 20.9 | 20.01 |
|  | 3931.71 | 0.0293 | 25.38 | 29.66 | 5.25 | 6.62 |
|  | 4614.25 | 0.0354 | 8.58 | 10.62 | 2.5 | 3.69 |
|  | 4362.64 | 0.0388 | 12.84 | 15.86 | 3.97 | 5.5 |
|  | 3365.55 | 0.0388 | 17.19 | 19.68 | 4.3 | 4.61 |
|  | 4396.17 | 0.0388 | 10.39 | 12.85 | 5.44 | 4.99 |
|  | 2899.53 | 0.0447 | 27.2 | 44.36 | 9.79 | 40.43 |
| CLBP vs. N | 5755.18 | 0.0000847 | 45.05 | 27.84 | 20.82 | 7.21 |
|  | 7062.96 | 0.0278 | 8.53 | 10.63 | 1.59 | 2.45 |
|  | 1450.69 | 0.0318 | 11.17 | 23.15 | 6.03 | 18.53 |
|  | 5629.16 | 0.0318 | 15.81 | 11.53 | 5.96 | 2.37 |
|  | 5132.41 | 0.0356 | 13.52 | 17.14 | 2.48 | 5.38 |

M/Z: The mass/charge characteristic of each Protein/ peptides;

*P* Value: *P* value of wilcoxon test (2class) or Kruskal-Wallis (>2 class);

Ave N: Peak area (intensity.) average of class N;

SDN: standard deviation of the peak area average of class N.
